# A Case of Fissure of the Cranium, *Attended with Apparent Extravasation, Successfully Treated without the Use of the Trephine; to Which Are Added, a Few Observations*

**Published:** 1803-09-01

**Authors:** 

**Affiliations:** Surgeon, *of New Surry Street, Blackfriars Road*


A CASE OF FISSURE OF THE CRANIUM, *attended with apparent Extravasation, successfully treated without the Use of the Trephine; to which are added, a few Observations. Communicated by* Mr. ALDIS, Surgeon, *of New Surry Street, Blackfriars Road.*

AS the brain is the seat of sensation, and consequently less able to resist an injury done to it, it is therefore more liable to be affected, and is attended with more danger from external violence than any other part of the body, the lungs perhaps excepted.

Much study and attention have been employed to ascertain the best means of affording relief in injuries of the head from external violence; yet many discoveries remain to be made before we can arrive at the acme of knowledge in this branch of the profession.

The only means of relief usually employed till of late years, for every species of injury done to the head, attended ed with fracture, fissure, or depression of a portion of bone, was the trephine, in order to raise the depressed part to its natural level, or to remove any matter that might be collected beneath the cranium and *dura mater*.

Surgeons, till very lately, were totally unacquainted with the good effects which would accrue from omitting the operation of perforating the cranium; an operation which I should think every judicious practitioner must admit of being attended with considerable danger in any part of the head, but more particularly so in the course of the sinuses, that it was not till within these few years, comparatively speaking, that patients were known to recover without the assistance of the trephine, in cases of fracture, attended with all the alarming symptoms of sickness, vertigo, stertorous breathing, dilated pupils, &c. &c.

Among those who are against the too frequent use of the trephine, may be reckoned Mr. John Bell, and particularly Mr. Abernethy, whose abilities and extensive practice rank him among the first of those who have declaimed against this practice. Indeed, it was principally from the perusal some few years since of a truly valuable publication of his, entitled, an Essay on Injuries of the Head, which first gave rise to the opinion I now entertain. He there points out several dreadful cases which were successfully treated by bleedings, and pursuing the anti-phlogistic regimen, without having recourse to any operation.

You will not infer from what I have written, that there are no cases which occur wherein I suppose the assistance of the trephine would be unnecessary; by no means. What I would wish to inculcate is, that I firmly believe a great number of fractures of the cranium, attended with depression or extravasation, would do well without the use of the trephine, which is often unnecessarily applied. From all the accounts which I have ever heard of, and I have taken some pains to ascertain the surgical treatment of injuries of this kind done to the head, the trephine for the most part, nay, almost always, has been used. I think I may venture to assert therefore that, especially among country medical gentleman, this instrument is frequently had recourse to, when, had they pursued the same treatment which I have done in the following case, they would, in all probability, have been equally as successful.

On the tenth of June last, I was requested to visit Thomas Cann, a servant of Mr. Gill, of New Surry Street, who I was told had just received a kick from a horse. On my arrival, I found him lying on a bed to all appearance lifeless;

 but but on taking hold of his hand, I could just feel his pulse, which beat small and irregular; at this instant he started, and continued to breathe with great difficulty and stertor. The pupils were dilated, and sickness ensued. Examining his head, a wound was discovered to be partly on the frontal, parietal, and temporal bones, which could be distinctly seen by the division of the integuments to the extent of four inches and an half, with the cranium denuded an inch every way. The fissure appeared to be about an inch and an half in length. The wound I immediately cleaned with a sponge and some warm water, then brought the edges in contact, by making three sutures; and by means of slips of sticking plaster, endeavoured to promote adhesion, as also to prevent the access of air to the wound. This being done, and the part secured by a proper bandage, I asked him how he felt himself; but having no answer, I repeated my question in a more loud tone of voice, to which he was equally insensible. I then left him, and desired the nurse not to give him any thing but barley water or a little tea, and that I should again visit him in the space of two hours. On repeating my visit, I found, what I expected, that a re-action of the pulse had taken place, and that it was much stronger than when first I saw him. The sickness had considerably abated, his respiration less stertorious; and he answered, though with great reluctance, any question which was put to him. I now copiously bled him, and sent him a saline mixture to take, as also the next morning ordered him a cathartic.

11th, I found my patient quite relieved from those symptoms which before had distressed him; that what remained now was only a smarting of the wound, and a degree of pain on the occiput.

12th, As the pain of the head continued, and his pulse permitted it, I repeated the bleeding and cathartic, and continued the mixture and same spare diet as at first.

13th, This morning he was not so well as yesterday. He had some degree of fever, and also complained more of his head. I did not in this instance again bleed him, but ordered him three grains of the pulv. antimon. to be taken every three or four hours with the saline mixture. I now thought proper to remove the dressings and examine the wound, which, to my great satisfaction, was in a state of doing extremely well. On removing the plasters, a small discharge of healthy pus issued from the wound, the surrounding scalp appeared free from inflammation, mation, and had perfectly united to the cranium. The adhesive dressings were repeated.

14th, The powders had brought on a gentle perspiration, and greatly relieved the febrile symptoms of yesterday.

15th, The fever being subsided, and as he expressed a wish to leave his bed, I told him, if he remained so well till the next day he should be permitted to rise, as also to have his diet a little altered.

16th, As every thing went on agreeably to my wishes, my patient left his bed; I repeated his cathartic, omitted the antimonial powders, and renewed the dressings.

17th, Every circumstance bade fair for a speedy recovery: all pain had left him, he slept well, was free from fever. In short, nothing now remained but a desire of the patient to follow his usual occupation, which latter could not at present be complied with; but I requested him to come to my house every other morning, to have his head dressed; which he did, and continued regularly so to do till the second of July, when it was perfectly healed.

Although in the preceding case the lancet was only used twice, as circumstances did not require a repetition; yet, should there be any apprehension of great inflammation, I would recommend venesection to be used more frequently at the very commencement of the complaint, as the effects would be much greater in checking the inflammatory symptoms; for when it is deferred to a later period, the patient would be too low to admit of such active means, and his recovery from such a state would be rendered extremely precarious. While the absorption of the extravasated matter is going on, pain or stupor will continue more or less possibly; but not so as to excite alarm, unless in particular circumstances. The same space of time generally elapses before the brain can accommodate itself to its new situation, in cases of depressed portions of bone, during which the same symptoms may be expected to occur.

Should it be objected by some, that the above was not a case of extravasation, but merely of simple concussion of the brain; to such objections I could only reply, that as those symptoms occurred, which are evident marks to the contrary, such as stertor, dilatation of the pupils, &c. &c. I was led to suppose otherwise.

It is now known, and I hope soon it will be generally so, that the brain will retain its functions when considerably compressed, without injury to the person; but to what degree it is perhaps difficult to say, as it appears to be

 more more affected in some constitutions than in others, according to the susceptibility of that organ, which varies in different subjects. Several cases are related by authors, of the brain accommodating itself to portions of bone when considerably beaten in. Others again have had cases of depressed portions of bone in young subjects, when they have of themselves regained their natural level, and what extravasated blood there was soon became absorbed after it was rendered fit to be taken up, by having undergone a chemico-animal process.

If by mentioning the above medical gentlemen, whose opinions go to corroborate what I have asserted, and by relating the above case, it should in any degree tend to arrest the practice which I have attempted to oppose, this little paper will have amply answered its intended purpose.

